# A 25-year Experience in Quality Assurance: A Methodological Review of Laboratory Quality Control and Quality Assurance in the Tehran Lipid and Glucose Study (TLGS)

**DOI:** 10.5812/ijem-168018

**Published:** 2026-08-16

**Authors:** Mehdi Hedayati, Maryam Tohidi, Iraj Azimzadeh, Amir Abbas Momenan, Maryam Sadat Daneshpour, Maryam Zarkesh, Safura Pakizehkar, Samaneh Hosseinzadeh, Fereidoun Azizi

**Affiliations:** 1 Cellular and Molecular Endocrine Research Center, Research Institute for Endocrine Molecular Biology, Research Institute for Endocrine Sciences, Shahid Beheshti University of Medical Sciences, Tehran, Iran; 2 Prevention of Metabolic Disorders Research Center, Research Institute for Endocrine Sciences, Shahid Beheshti University of Medical Sciences, Tehran, Iran; 3 Endocrine Research Center, Research Institute for Endocrine Disorders, Research Institute for Endocrine Sciences, Shahid Beheshti University of Medical Sciences, Tehran, Iran

**Keywords:** Quality Control, Quality Assurance, Clinical Laboratory Techniques, Longitudinal Studies, Cohort Studies, Lipids, Glucose, Tehran Lipid and Glucose Study (TLGS)

## Abstract

**Context:**

Quality control (QC) and quality assurance (QA) are critical for validating laboratory data in longitudinal studies. The Tehran Lipid and Glucose Study (TLGS), the first large cohort study in Iran, was designed to investigate the incidence of and risk factors for cardiovascular diseases. This article reviews the methodological quality control system used for biochemical testing in the TLGS, with an emphasis on its design, implementation, evaluation, and data stability across study phases.

**Evidence Acquisition:**

In this methodological review, internal and external quality-control protocols used in phases 2 to 7 of the TLGS were analyzed and compared with international standards, including those of the Cardiovascular Health Study and the Atherosclerosis Risk in Communities study. Analytical precision data (coefficient of variation [CV%]) for glucose, creatinine, total cholesterol, triglycerides, HDL-C, ALP, ALT, and AST were extracted from a central database, and temporal trends were evaluated. Internal quality control was performed daily using two levels of control sera (normal and abnormal), and interlaboratory accuracy was assessed through participation in an external evaluation program.

**Results:**

The mean CV for all tests across the study phases was less than 5% (glucose, 1.95%; creatinine, 3.10%; cholesterol, 2.05%; triglycerides, 2.16%; HDL-C, 3.33%; ALP, 2.10%; ALT, 3.00%; and AST, 3.35%). External quality-control results received an “Excellent” rating for all parameters. The temporal trend in CVs demonstrated stability and a gradual improvement in precision across phases. Levey–Jennings charts, Westgard rules, and weighted regression methods were effective in detecting systematic deviations and enabling prompt corrective action.

**Conclusions:**

Establishing a multilevel QC/QA system in the TLGS ensured the stability and accuracy of biochemical data in a large national cohort. The findings indicate that the accuracy of biochemical testing was consistent with international standards and that the resulting data are highly reliable for epidemiological and clinical analyses. This experience may serve as a local model for other longitudinal studies in developing countries.

## 1. Context

The accuracy and validity of laboratory data are paramount in longitudinal epidemiological studies because they directly determine the reliability and generalizability of scientific findings (1). In cohort studies examining associations between risk factors and disease incidence over time, even minor laboratory errors can bias relative risk estimates, reduce statistical power, and ultimately lead to incorrect conclusions (2). Therefore, a comprehensive quality control (QC) and quality assurance (QA) system is essential throughout the testing process, from preanalytical sampling to postanalytical data reporting (3).

Large international studies, such as the Atherosclerosis Risk in Communities (ARIC) study (4), the Cardiovascular Health Study (CHS) (5), and the Framingham Heart Study (FHS) (6), have provided successful models for multilevel quality-control systems. Their methods are well documented, primarily in high-resource settings. Central laboratories, standardized protocols for blood collection, transport, and storage, and participation in External Quality Assessment (EQA) programs have substantially reduced random and systematic errors (4-6). These studies also illustrate the long-term evolution, challenges, and practical adaptation of quality systems in large-scale research. However, most published QC/QA frameworks originate from settings with stable infrastructure, uninterrupted access to laboratory supplies, and adequate financial and technical resources. Maintaining analytical consistency for decades in developing countries poses additional challenges, including resource constraints, equipment maintenance requirements, reagent-lot variability, and limitations in laboratory infrastructure. Evidence from resource-limited settings, including the Middle East, remains limited. Therefore, region-specific approaches to maintaining data integrity over decades are important to the global scientific community.

In Iran, the need for an indigenous model for long-term monitoring of metabolic and cardiovascular indices led to the Tehran Lipid and Glucose Study (TLGS). Initiated in 1999 by the Endocrine and Metabolism Research Center of Shahid Beheshti University of Medical Sciences, the TLGS was the first large prospective study in the country designed to identify and follow risk factors for cardiovascular diseases, diabetes, and lipid disorders in the urban population of Tehran. The study recruited more than 15,005 participants aged 3 to 69 years and used a baseline-and-follow-up design to examine changes in blood glucose and lipids and related clinical outcomes over time (7). The large population, long follow-up, and range of biochemical tests, including glucose, lipids, and liver enzymes, required a precise and continuous QC/QA system based on international standards (8). Because these parameters are key indicators for diagnosing diabetes and dyslipidemia, strict quality control at the preanalytical, analytical, and postanalytical stages is essential for maintaining reliable results (9). Errors at any stage, including freezer-temperature fluctuations, sample hemolysis, or incorrect instrument calibration, can substantially affect longitudinal data and temporal trends (10).

The TLGS internal and external quality-control system includes daily monitoring of instrument performance, normal and abnormal control sera, Levey–Jennings charts, multiple Westgard rules, and participation in a national external evaluation program, thereby providing a coherent framework for data quality assurance (11). Together with ongoing staff training, accurate documentation, and analysis of coefficient-of-variation (CV%) trends across study phases, this system has enabled the long-term evaluation of laboratory-data stability (12).

Accordingly, this methodological review of the TLGS quality-control system reflects the scientific and technical maturity of the national project and may provide a model for designing and implementing similar programs in other countries, particularly in resource-limited settings (7).

This article reviews the TLGS QC/QA framework over its 25-year history. We evaluate the structure, methods, and performance indicators of the system and compare its evolution with international benchmarks. The goals are to demonstrate the reliability of TLGS data and to provide a replicable model and practical lessons for sustainable quality-assurance systems in similar long-term studies.

## 2. Evidence Acquisition

### 2.1. Study Design and Quality Control Framework

The Tehran Lipid and Glucose Study (TLGS) was initiated as a prospective cohort study in 1999 and has been conducted in several follow-up phases. Because QC data from phase 1 are no longer accessible, this review covers phases 2 to 7 (2002 - 2025), during which the main biochemical tests, including glucose, creatinine, total cholesterol, triglycerides, HDL-C, ALP, ALT, and AST, were performed centrally at the TLGS reference laboratory (7). To ensure the accuracy and precision of laboratory data, a comprehensive QC/QA system was designed and continuously implemented. The system covered three main stages: 1) preanalytical control of sampling, sample transport, and storage; 2) analytical control of instruments, kits, and operators; and 3) postanalytical control of data entry, result reporting, and statistical performance trends (13). The TLGS QC/QA system was developed in accordance with ISO 15189:2022 standards and Good Laboratory Practice principles and was periodically reviewed and evaluated.

### 2.2. Definitions of Key Quality Control and Analytical Terminology

Evaluation and discussion of laboratory data quality require specific, clearly defined terminology. To ensure clarity and consistency and to avoid ambiguity, key concepts related to analytical performance, including accuracy, precision, bias, and total analytical error, are defined in Table 1.

**Table 1.** Definitions of Key Quality-Control Terminology

Terms	Definition	Related Concept / Note
**Accuracy**	The closeness of a measured value to the true value	Primarily affected by systematic error (bias)
**Precision**	The closeness of repeated measurements to each other	A measure of random error. Expressed as CV% or standard deviation
**Bias**	The difference between the average of measured values and the true value	A measure of systematic error. Determined by comparing to a reference method or target value
**Random error**	Unpredictable fluctuations in measurements that cause a spread of results around a mean	Affects precision. Cannot be corrected, only minimized
**Systematic error**	A consistent, directional error that causes all measurements to be consistently higher or lower than the true value	Affects accuracy. Can be identified and corrected (e.g., through calibration)
**Coefficient of Variation (CV%)**	A standardized measure of precision, expressed as a percentage: (standard deviation / Mean) × 100	Allows comparison of precision across tests with different units or means
**Repeatability**	Precision under the same operating conditions over a short time (same lab, same analyst, same equipment)	Also known as within-run precision
**Reproducibility**	Precision under different conditions (e.g., different days, different analysts, different labs)	A broader measure of precision than repeatability
**Total analytical error (TE)**	The total error of an analytical test, combining both random and systematic components	The most common formula is: TE = \|Bias\| + (1.96 × CV%). It reflects the worst-case error for a single patient result
**Technical error (TE)**	A measure of the random variation or imprecision between replicate measurements of an identical sample	Primarily reflects random error; it is a measure of imprecision and a key component of total error
**Reliability**	The consistency of a measure; in a lab context, often used interchangeably with precision or reproducibility	A reliable test yields consistent results on repeated trials
**Validity**	The extent to which a test measures what it claims to measure	In a lab context, closely related to accuracy and the absence of analytical interferences

### 2.3. Sampling and Data Collection

Sampling was performed in all phases by trained and certified personnel. Blood was drawn in the morning after a 12-hour fast. Two types of samples were prepared:

• Clot sample (7 cc): for serum preparation and biochemical tests.

• EDTA-anticoagulated sample (3 mL): for future molecular-genetic studies and storage in the biobank.

Blood was collected using a 21G needle and a vacuum system (Vacutainer), with tourniquet time kept below 1 minute. Each tube had a unique identification code to enable sample tracking throughout the process. After 15 minutes at room temperature, samples were centrifuged (Eppendorf 5810R) at 3000 rpm for 10 minutes. The separated serum was aliquoted into four 0.5 cc microtubes and stored at -80 °C in an Ultra Freezer (Sanyo MDF-U73V). Freezer temperature was recorded daily, and an automatic alarm system was used to issue a notification if the temperature increased by more than 5 °C.

### 2.4. Biochemical Tests

The specific methods for all biochemical measurements are detailed in Table 2. All biochemical analyses were performed using Pars Azmoon reagent kits (Pars Azmoon Co, Tehran, Iran) on a Selectra Pro M automated chemistry analyzer (Vital Scientific, Netherlands), which was calibrated daily. Whenever a new reagent lot was introduced, calibration verification and parallel quality-control assessment were performed before routine use to maintain analytical continuity across study phases.

**Table 2.** Detailed Methodology for Biochemical Measurements

Test Types	Test Method	Unit of Measurement
**Glucose**	Glucose Oxidase	mg/dL
**Creatinine**	Modified Jaffe or Kinetic Method	mg/dL
**Total Cholesterol**	Enzymatic (CHOD–PAP)	mg/dL
**Triglyceride**	Enzymatic (GPO–PAP)	mg/dL
**HDL-C**	Homogeneous direct assay based on antibody-mediated masking	mg/dL
**AST and ALT**	IFCC Method without Pyridoxal	U/L

Daily checks included the washing mechanism, the sample pump, and reagent temperature.

### 2.5. Internal Quality Control

Internal quality control (QC) was performed every working day before study samples were analyzed. For each parameter, two serum-control levels (normal and abnormal; Pars Azmoon) were used. Control results were recorded on monthly Levey–Jennings charts, and multiple Westgard rules (1_2s, 2_2s, R_4s, 4_1s, and 10_x) were applied to interpret deviations (14, 15). If a deviation outside the acceptance range (± 2 SD or ± 3 SD) was observed, the instrument was recalibrated, the control solution was replaced, and the test was repeated when necessary. Control data were accumulated monthly, and the coefficient of variation (CV%) was calculated as the key internal QC metric for monitoring analytical precision.

All CVs were less than 5%, indicating high analytical precision according to Clinical Laboratory Standards Institute (CLSI) criteria (16).

### 2.6. External Quality Assessment

To ensure interlaboratory standardization, the laboratory participated in a quarterly External Quality Assessment (EQA) program coordinated by the Iranian Pioneer Quality Control Center (Tehran, Iran). Performance for key biochemical analytes (glucose, creatinine, cholesterol, triglycerides, HDL-C, ALP, ALT, and AST) was assessed by comparing results from blinded samples with established target values. Under the EQA program, a result was considered acceptable when it fell within ± 2 standard deviations (SD) of the target value. TLGS laboratory performance was ranked using Z-scores according to criteria established by the EQA scheme (7).

In selected cycles, blind replicates of real samples were retested to estimate repeatability. The mean correlation coefficient between replicate biochemical-test results was greater than 0.94, indicating very good reproducibility.

#### 2.6.1. Interlaboratory Accuracy Assessment

In selected analytical cycles, a subset of real samples was analyzed in blind duplicate form to evaluate method repeatability and interlaboratory accuracy. Technical Error of Measurement (TEM) was used as an indicator of analytical repeatability and was calculated from duplicate analyses. In addition, total analytical error (TAE) for each biochemical measurement was estimated using the root-sum-of-squares approach, which combines random and systematic error.

TE was calculated according to established method-validation guidelines (17) using the following equation:

$$\mathrm{TE}=\sqrt{{\left({\mathrm{CV}}_{\mathrm{mean}}\right)}^{2}+{\left({\mathrm{Bias}}_{\mathrm{abs}}\right)}^{2}}$$

Blind replicate testing demonstrated high reproducibility across the biochemical assays.

#### 2.6.2. Total Error Clinical Assessment

To provide a comprehensive clinical assessment of analytical accuracy, TAE was calculated for each analyte. TAE combines imprecision, measured by CV%, and systematic bias, determined from the External Quality Assessment program, into a single value for comparison with clinical quality requirements.

The components of total analytical error were determined according to the CLSI EP15-A3 guideline for verification of precision and bias. TAE for each analyte was then calculated using the standard formula:

Total analytical error = |Bias| + (1.65 × CV%)

The observed values were compared with TAE goals based on desirable analytical performance specifications derived from biological variation and reported in standard clinical chemistry references.

### 2.7. Equipment Monitoring and Sample Storage

Temperatures of freezers, refrigerators, and centrifuges were recorded daily, and the data were stored in the laboratory software system (LIMS QC Module v3.2). If the freezer temperature rose above -80 °C, the automatic alert system notified the technical manager by SMS. Equipment was checked monthly for centrifuge speed, incubator temperature, and pipette accuracy, and periodic calibration certificates were recorded every 6 months.

### 2.8. Data Control and Data Quality Assurance

Data from the autoanalyzer were transferred directly to a central database and reviewed by a senior QC expert. Values outside physiological limits or suspected entry errors were confirmed or corrected by re-examining the sample. Data were backed up automatically each day. The QA system included quarterly internal audits, SOP documentation reviews, and annual staff retraining. At each audit, performance checklists were evaluated using the following indicators:

• Percentage of samples acceptable under internal QC.

• Test turnaround time.

• Percentage of samples rejected because of hemolysis or insufficient volume.

• Interphase CV%.

Turnaround time (TAT) was defined as the interval from sample receipt in the laboratory to reporting of the final verified result.

### 2.9. Statistical Analysis and Trend Assessment

For each parameter in each phase, the mean, standard deviation, and CV% were calculated. Weighted linear regression was applied to monthly control averages to examine changes in precision over time. CUSUM was calculated in an Excel spreadsheet using the target value for each analyte, the observed control value for each run, and the corresponding standard deviation (SD). The allowance parameter (k) was set to 0.5 × SD, and the decision interval (h) was set to 5 × SD, in accordance with standard quality-control guidelines. A signal was generated when the cumulative sum exceeded the upper or lower control limit, indicating a potential out-of-control condition requiring investigation. P < 0.05 was considered statistically significant.

### 2.10. Research Ethics and Considerations

The TLGS biochemical and quality-control protocols were approved by the Research Ethics Committee of Shahid Beheshti University of Medical Sciences. All participants provided written informed consent, and their information was kept confidential and coded.

## 3. Results

### 3.1. Assessment of Data Completeness and Reliability (Repeatability)

During follow-up phases from 2002 to 2015, data for the main biochemical tests, including glucose, creatinine, total cholesterol, triglycerides, HDL-C, ALP, ALT, and AST, were recorded completely and reliably for more than 95% of collected samples. The overall rate of data deletion due to hemolysis or analytical error was less than 2%. This high level of completeness supports the reliability and repeatability of the measurement process over time. In selected cycles, blind replicates of real samples were retested. The mean correlation coefficient between initial and replicate biochemical test values was greater than 0.94, indicating very good reproducibility. These findings support the robustness of the preanalytical and analytical procedures.

Table 3 summarizes the coefficients of variation (CV%) obtained from internal quality control for each parameter across study phases.

**Table 3.** Summary of Long-Term Internal Quality-Control Performance (Coefficient of Variation, CV%) Across TLGS Phases 2 to 7 ^a^

Analyte / Phase	Phase II	Phase III	Phase IV	Phase V	Phase VI	Phase VII	CLSI Acceptance Range
**Glucose (Glu)**	2.3 - 2.6	2.1 - 2.5	1.5 - 2.0	1.5 - 2.0	1.4 - 1.8	1.6 - 2.1	< 5
**Creatinine (Cre)**	1.7 - 3.1	3.0 - 4.5	2.9 - 5.0	1.9 - 3.3	1.9 - 2.8	2.3 - 4.8	< 5
**Cholesterol (Chol)**	1.7 - 3.1	1.9 - 2.2	1.8	1.7 - 2.0	1.5 - 2.2	1.6 - 3.1	< 5
**Triglycerides (Tg)**	2.0 - 2.1	1.9 - 2.4	1.8 - 2.5	2.0 - 2.4	1.8 - 3.0	1.5 - 2.5	< 5
**HDL-C**	3.0	5.0	2.9	2.45 - 4.6	2.16 - 2.35	2.6 - 4.0	< 5
**ALP**	N/A	N/A	N/A	N/A	N/A	1.7 - 2.5	< 5
**ALT**	N/A	N/A	N/A	N/A	N/A	1.1 - 4.9	< 10
**AST**	N/A	N/A	N/A	N/A	N/A	1.8 - 4.9	< 10

^a^ Values are expressed as percentage.

**Figure 1.**
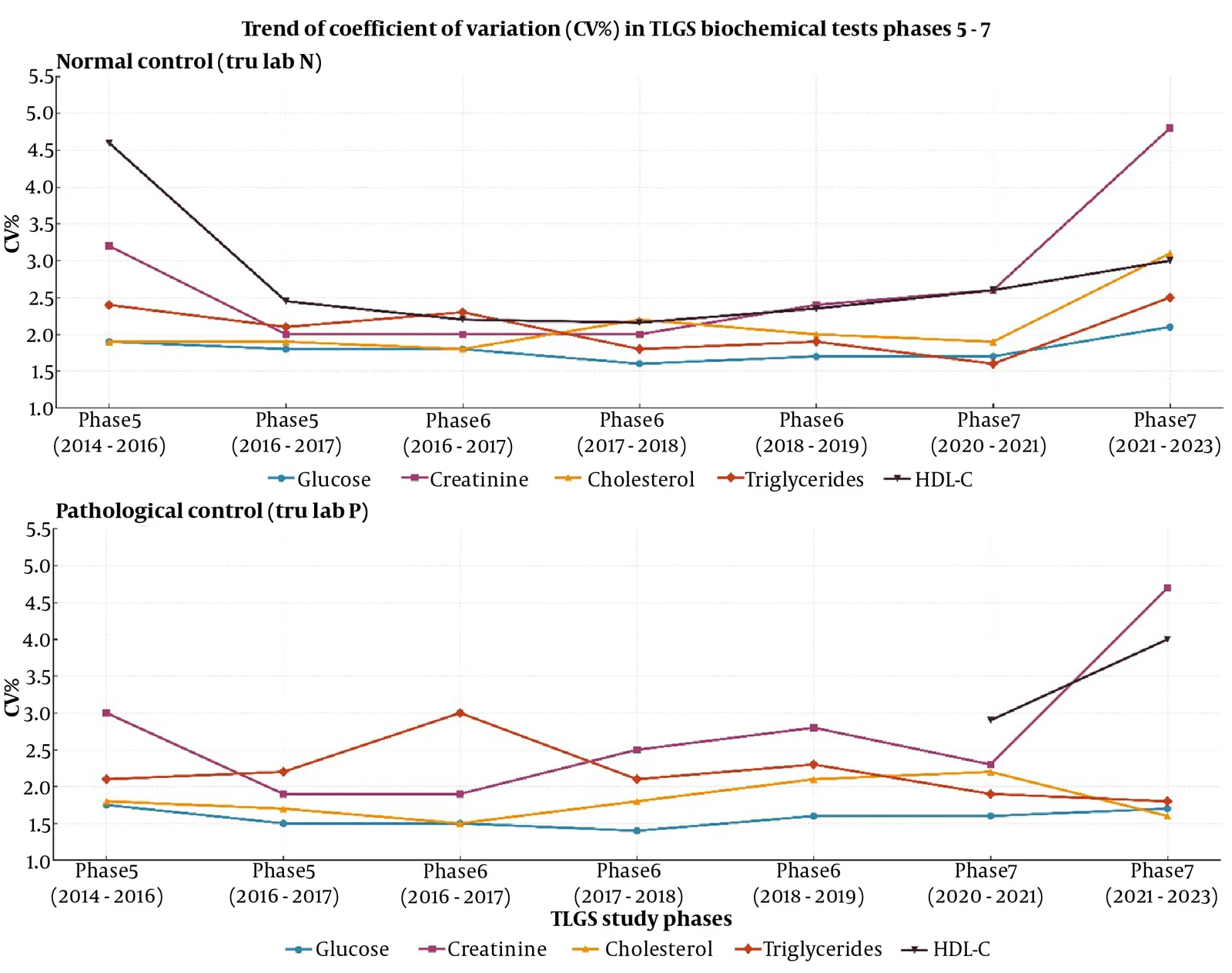
Trend in coefficient of variation (CV%) of biochemical tests across TLGS phases

As shown in Figure 1, CV% declined consistently for most analytes across successive study phases, indicating progressive improvement in analytical precision and long-term laboratory stability. All parameters were within the CLSI acceptable range (CV < 5%). This temporal trend in CVs indicates that mean random error decreased over time and reached substantial analytical stability.

### 3.2. Assessment of Analytical Accuracy (External QA)

During all External Quality Assessment (EQA) periods from 2002 to 2015, the mean deviation of TLGS results from the national average was less than 3%. Table 4 presents the interlaboratory accuracy assessment for the main parameters.

**Table 4.** TLGS External Quality Assurance Results Compared with the National Average

Parameter	Average Deviation from Target Value (Bias%)	Quality Score (Z-Score)	Evaluation Rank	Status
**Glucose**	1.2	0.35	Excellent	Accepted
**Creatinine**	-0.8	-0.15	Excellent	Accepted
**Total cholesterol**	0.7	0.12	Excellent	Accepted
**Triglyceride**	3.1	0.65	Excellent	Accepted
**HDL-C**	-4.2	-0.89	Excellent	Accepted
**ALP**	2.1	0.25	Excellent	Accepted
**ALT**	-1.5	-0.30	Excellent	Accepted
**AST**	0.6	0.10	Excellent	Accepted

According to International External Quality Assessment Scheme criteria, Z-scores below 2 indicate acceptable performance; in this study, all parameters were at the excellent performance level (Z < 1). The small deviations from EQA target values indicate minimal systematic bias and support the high accuracy of the analytical methods.

#### 3.2.1. Evaluation of Repeatability and Technical Error

To assess repeatability, 5% of random samples from each phase were retested in duplicate under blinded conditions. Correlations between initial and replicate biochemical values ranged from 0.94 to 0.98. Technical error (TE) was calculated and is presented in Table 5. All TE values were less than 5%, indicating good reproducibility and tight control of analytical processes.

**Table 5.** Technical Errors of Tests Across Study Phases ^a^

Parameters	Phase-II	Phase-III	Phase-IV	Phase-V	Phase-VI	Phase-VII	Total Mean TE
**Glucose**	2.52	2.38	2.01	1.98	1.75	2.05	2.11
**Creatinine**	3.22	4.82	5.10	3.47	2.87	4.85	4.06
**Total Cholesterol**	3.12	2.21	2.01	2.02	2.23	3.15	2.46
**Triglycerides**	2.11	2.44	2.52	2.41	3.02	2.52	2.50
**HDL-C**	3.00	5.00	2.90	4.62	2.28	4.02	3.64
**ALP**	N/A	N/A	N/A	N/A	N/A	2.55	2.55
**ALT**	N/A	N/A	N/A	N/A	N/A	4.91	4.91
**AST**	N/A	N/A	N/A	N/A	N/A	4.92	4.92

^a^ Values are expressed as percentage.

#### 3.2.2. Evaluation of Repeatability and Total Analytical Error

To assess repeatability, 5% of random samples from each phase were retested as blinded duplicates. Correlations between initial and replicate biochemical values ranged from 0.94 to 0.98, demonstrating excellent reproducibility.

To provide a comprehensive clinical assessment of analytical accuracy, TAE was calculated for each key analyte by combining imprecision (CV%) and bias. As shown in Table 6, TAE values for most tests, including glucose, met stringent clinical analytical goals, supporting the high quality and reliability of the data.

**Table 6.** Comparison of TLGS Laboratory Performance with Clinical Analytical Goals ^a^

Parameters	Our Imprecision (CV%)	Our Bias	Our Total Error ^b^	Clinical Goal For CV% ^c^	Clinical Goal for Bias ^c^	Clinical Goal for Total Error ^c^	Meets Clinical Goal?
**Glucose**	1.95	1.2	4.42	≤ 2.8	≤ 2.34	≤ 6.96	Yes
**Creatinine**	3.10	-0.8	5.92	≤ 2.98	≤ 3.96	≤ 8.87	Yes
**Total cholesterol**	2.05	0.7	4.08	≤ 2.98	≤ 4.1	≤ 9.01	Yes
**Triglyceride**	2.16	3.1	6.66	≤ 9.95	≤ 9.57	≤ 25.99	Yes
**HDL-C**	3.33	-4.2	9.69	≤ 3.65	≤ 5.61	≤ 11.63	Yes
**ALP**	2.10	2.1	5.57	≤ 3.23	≤ 6.72	≤ 12.04	Yes
**ALT**	3.00	-1.5	6.45	≤ 9.7	≤ 11.48	≤ 27.48	Yes
**AST**	3.35	0.6	6.13	≤ 6.15	≤ 6.54	≤ 16.69	Yes

^a^ Values are expressed as percentage.

^b^ TAE was calculated using the standard formula: |Bias| + (1.65 × CV %).

^c^ Clinical goals are based on desirable analytical specifications for clinical use derived from biological-variation data and the Westgard QC database.

### 3.3. Time Trend Assessment of Analytical Precision

Weighted linear regression of CV trends across phases showed negative, statistically significant slopes for all parameters (P < 0.05), indicating gradual improvement in test precision over time. For example, the CV for glucose decreased from 2.3% in phase 2 to 2.0% in phase 4, corresponding to a relative 13% reduction in analytical error. The greatest improvement was observed for triglycerides (15% reduction), probably due to optimization of the enzymatic method and tighter control of preanalytical processes.

### 3.4. Monitoring Systematic Deviations and Corrective Actions

Levey–Jennings charts and Westgard rules were used to monitor systematic deviations. On 4.2% of working days, at least 1 rule, usually 1_2s or 2_2s, was violated. Investigations showed that errors were mainly attributable to minor fluctuations in pipette volume or laboratory temperature. After corrective actions, including recalibration and repeat testing, values returned to the permissible range. No result was entered into the TLGS database after a severe Westgard-rule violation (1_3s or R_4s) without re-verification.

### 3.5. Data Control and Quality Audit

Quarterly QA audits estimated the data-entry error rate at less than 0.5% and the discrepancy rate between recorded data and raw sheets at less than 0.2%. Annual audits showed greater than 99% data consistency between phases. Mean turnaround time (TAT) for biochemical tests decreased from 72 hours in phase 2 to 48 hours in subsequent phases (P < 0.01).

### 3.6. International Comparison of QC/QA Performance

Comparison of mean CV% values in TLGS with those reported in major international studies showed equivalent or superior performance for most parameters (Table 7).

**Table 7.** Comprehensive Comparison of Analytical Performance in Major International Cohort Studies ^a^

Cohort Study	Glucose			Total Cholesterol			Triglycerides			HDL-C			Creatinine			ALP			ALT			AST		
	CV	Bias	TE	CV	Bias	TE	CV	Bias	TE	CV	Bias	TE	CV	Bias	TE	CV	Bias	TE	CV	Bias	TE	CV	Bias	TE
**TLGS (present study)**	1.95	1.2	4.42	2.05	0.7	4.08	2.16	3.1	6.66	3.33	-4.2	9.69	3.10	-0.8	5.92	2.10	2.1	5.57	3.00	-1.5	6.45	3.35	0.6	6.13
**Cushman et al. (5)**	1.86	NR	NR	2.52	NR	NR	2.62	NR	NR	3.56	NR	NR	3.58	NR	NR	NR	NR	NR	NR	NR	NR	NR	NR	NR
**Balık and Gücel (18)**	2.93/ 2.77	1.53	6.36/ 6.10	3.08/ 2.17	1.15	6.23/ 4.72	4.12/ 4.26	2.95	9.75/ 9.98	4.91/ 4.83	2.69	10.8/ 10.66	NR	NR	NR	8.02/ 6.71	2.28	15.51/ 13.35	3.55/ 3.20	1.87	7.72/ 7.15	3.82/ 4.04	2.81	9.11/ 9.47
**Verma et al. (19)**	2.74/ 2.04	3.66	8.18/ 7.03	2.86/ 2.66	5.08	9.8/ 9.47	4.74/ 3.01	7.41	15.23/ 12.38	3.69/ 3.62	-17.4	-11.3/ -11.4	5.42/ 3.22	-8.01	0.93/ -2.7	3.27/ 2.75	-2.56	2.84/ 1.98	4.75/ 3.23	12.7	20.54/ 18.03	4.77/ 3.11	13.2	21.07/ 18.33
**Çakmak et al. (20)**	2.0/ 1.9	3.5/ 2.8	6.9/ 5.8	1.2	0.03	2.0	3.3/ 3.5	5.0/ 0.8	10.5/ 6.6	2.1	0.03	3.4	2.1	3.5	7.0	4.7	4.6	12.4	3.0/ 1.6	6.33/ 1.5	11.4/ 4.1	2.6	8.3	12.6
**Lee et al. (21)**	1.7	NR	NR	1.7	NR	NR	4.5	NR	NR	NR	NR	NR	4.5	NR	NR	NR	NR	NR	NR	NR	NR	NR	NR	NR

^a^ Values are expressed as percentage. Abbreviation: NR, not reported in the source publication.

This comparison indicates that the TLGS QC/QA system achieved accuracy equivalent to or better than that reported in similar international studies, supporting the high quality of data generated in this national project.

## 4. Discussion

The results of this methodological review show that the QC/QA system designed for the Tehran Lipid and Glucose Study (TLGS) is consistent with internationally accepted standards for large cohort studies in terms of structure, continuity, and performance. Low and stable coefficients of variation across biochemical tests, excellent ratings in external quality-control programs, and decreasing analytical error over follow-up provide strong evidence that the QC/QA system effectively maintained the accuracy and integrity of TLGS laboratory data (7).

From an epidemiological perspective, maintaining low analytical variability is more than a laboratory accomplishment. Measurement error can attenuate associations between exposure variables and disease outcomes and reduce the statistical power of longitudinal analyses. Therefore, the sustained analytical precision observed in TLGS increases confidence in risk estimates and temporal-trend analyses throughout the study period.

The QC/QA system designed for the TLGS is consistent with internationally accepted standards. Maintaining stable CV% values of approximately 2% - 3% across biochemical tests is particularly important in a longitudinal study, in which distinguishing true metabolic changes from analytical drift is a fundamental challenge (22). By minimizing technical variance, the TLGS QC/QA system helps ensure that observed trends reflect biological phenomena rather than analytical instability. Maintaining a homogeneous analytical platform across study phases is consistent with rigorous approaches used in other major cohort studies, including ARIC and CHS, and supports the reliability of TLGS data (4, 5).

Reducing analytical imprecision increases statistical power to detect true trends by reducing variance attributable to measurement error. Accordingly, the low analytical imprecision achieved in TLGS improves the ability to detect true metabolic changes at the population level (12, 23). This stability is particularly important because biochemical variables such as glucose, triglycerides, and HDL-C serve as primary exposures and outcomes in numerous TLGS analyses. Reducing analytical noise minimizes regression-dilution bias and improves the detection of genuine biological changes over time.

### 4.1. Internal Analysis

The TLGS laboratory follows standard internal quality-control procedures recommended by international guidelines, including the use of normal and pathological control sera and monitoring through Levey–Jennings charts. Deviations from acceptance limits occurred on fewer than 5% of working days, indicating efficient and timely instrument calibration and adjustment. The application of multiple Westgard rules to accept or reject test results was intended to prevent type I errors (rejecting correct results) and type II errors (accepting incorrect results). International studies have shown that appropriate implementation of Westgard 1_2s, 2_2s, and R_4s rules can identify up to 80% of systematic errors before results are released. In TLGS, strict application of these approaches ensured that no outliers were included in the final database. Blind duplicates and pooled serum were also used to assess intra-assay and interassay precision, which is standard practice in longitudinal studies (24-26). The high correlation between initial and repeat results (r > 0.95) indicates excellent long-term analytical stability across technicians and highlights the importance of continuous training and equipment stability (TLGS Internal QC/QA Reports, unpublished data).

### 4.2. Interlaboratory Precision Assessment and International Comparison

Continuous participation of the TLGS laboratory in an External Quality Assessment (EQA) program supervised by the Pishgam Iranian Center allowed laboratory performance to be compared with national and international standards. Z-scores below 1 across all tests indicate very small deviations from assigned values and therefore high analytical accuracy. According to international EQA guidelines, Z-scores within ± 2 are generally considered satisfactory, and values closer to 0 indicate better analytical performance.

Compared with published quality-control data from large population-based cohorts, the CV% and total error values observed in the TLGS laboratory for total cholesterol and triglycerides fall within internationally accepted analytical performance limits and are similar to, and in some cases numerically lower than, those reported in major cohort studies such as ARIC and CHS (data not shown). This performance is likely related to the continued use of a fixed autoanalyzer model, a uniform kit brand, and regular calibration. In long-term cohort studies such as the Framingham Offspring Study, between-phase analytical variability can arise from changes in assay platforms, reagents, or reagent lots. In contrast, the consistent instrumentation and assay methods used throughout TLGS are an important strength that helps limit increases in CV% over time.

### 4.3. Analysis of Analytical Performance and Error Sources

The high analytical precision of the study, with CVs consistently below 3% and within the < 5% acceptance limit specified by CLSI EP15-A3, indicates stable performance of the instruments and kits. More importantly, the TLGS system addressed multiple sources of error. Analysis of total error showed that the combined effects of imprecision and bias met clinical goals, supporting the effectiveness of monitoring analytical and preanalytical processes. Preanalytical errors, such as delays between blood collection and centrifugation or temperature fluctuations during sample transport, can account for a large proportion of total error. In TLGS, strict adherence to the 15-minute precentrifugation interval and storage at -80 °C helped control this source of variation.

### 4.4. Role of the Quality Assurance System and Periodic Audits

QC focuses on routine process control and error correction, whereas QA addresses the entire quality system and emphasizes error prevention. At TLGS, a dynamic QA system that included quarterly internal audits, independent annual audits, and periodic technician retraining created an ongoing mechanism for maintaining system stability (27). Quarterly audits showed a data-entry error rate below 0.5%, suggesting that the digitization process and 2-step data-entry controls effectively limited secondary errors. The TLGS QA system extended beyond laboratory measurements to personnel training, documentation management, and laboratory supplies, an approach also used in advanced cohort studies (28).

### 4.5. Limitations and Challenges

Several challenges were identified despite favorable quality-control results. First, maintaining a stable supply of laboratory kits from a single brand was difficult in some years and required the use of different batches, potentially introducing minor inconsistencies between phases. Despite the robustness of the QC/QA framework, residual analytical variation cannot be completely excluded. Over more than 2 decades of follow-up, unavoidable changes in reagent lots, calibrator batches, maintenance procedures, and laboratory conditions may introduce subtle analytical shifts that are difficult to detect fully. Although internal and external quality-control procedures were designed to identify and correct such variation, small undetected biases remain an inherent limitation of long-term cohort studies.

Second, the lack of an international external evaluation program such as IFCC-EQA for some parameters limited direct global comparison. In addition, gradual wear of autoanalyzers during the final study phases could increase mechanical error; this possibility was managed through preventive maintenance and recalibration. From a study-design perspective, incomplete documentation of transport temperatures in early phases was a limitation identified in initial audits and was addressed later by adding an automatic temperature-recording system.

### 4.6. Scientific and Practical Implications

The consistent quality of TLGS biochemical data has enabled rigorous epidemiological analyses, metabolic risk modeling, and international comparative studies. The reliability of these data has supported numerous publications on diabetes, metabolic syndrome, and cardiovascular diseases. From a public-health perspective, the TLGS experience provides a local model for countries planning large-scale longitudinal studies, particularly in settings with limited resources and variable laboratory infrastructure. The simple but systematic TLGS QC/QA design showed that adherence to key principles—instrument stability, ongoing training, regular calibration, and careful documentation—can achieve a level of quality similar to that of advanced centers worldwide (7).

### 4.7. Overall Analytical Conclusion

Overall, the TLGS QC/QA system has 3 notable features:

• High temporal stability: CV and TE remained stable for more than a decade, with no evidence of gradual degradation in analytical precision.

• Conformity with international standards: Performance matched or exceeded that of the CHS and ARIC studies for precision and accuracy indicators.

• Comprehensive coverage: The quality system extended from blood collection and processing through result reporting and data recording.

Beyond demonstrating laboratory performance, this study provides a transferable framework for establishing sustainable QC/QA systems in long-term cohort studies in developing countries. Lessons from the TLGS may help emerging population-based studies balance scientific rigor with practical resource limitations.

### 4.8. Conclusion

Establishing a comprehensive quality control and quality assurance system in the Tehran Lipid and Glucose Study (TLGS) represents an important achievement in Iranian epidemiological research. This methodological evaluation indicates that accuracy and precision consistent with international standards can be achieved, despite infrastructure and equipment limitations, through careful protocol design, continuous personnel training, and periodic process monitoring. An average coefficient of variation (CV%) below 3% across biochemical tests, together with total analytical error meeting stringent clinical goals for key analytes such as glucose, demonstrates the efficiency of internal quality control and the stability of laboratory performance over more than a decade. Continuous participation in external quality-control programs, with excellent ratings for all parameters, supports measurement accuracy and data reliability. The gradual reduction in analytical error and improvement in turnaround time (TAT) across successive phases further indicate the system’s capacity for continuous improvement. From a methodological perspective, the TLGS experience suggests several principles for designing QC/QA systems in future cohort studies:

• Stability of the analytical platform and kit brand: Changes in instruments and consumables should be minimized to avoid introducing interphase variance.

• Continuous, focused monitoring: Maintaining a QC database and analyzing CV trends at regular intervals enable early detection of systematic deviations.

• Periodic training and evaluation of personnel: Maintaining technical proficiency over time is as important as maintaining equipment and consumables.

• Integration of internal and external quality control: Combining these levels provides the highest degree of data assurance.

• Thorough documentation and periodic audits: Documentation is essential for scientific accountability and maintaining quality.

The TLGS demonstrates that an effective QC/QA system in a large longitudinal project can be both feasible and sustainable. This experience may provide a valuable implementation model for national and regional projects in metabolic epidemiology. Because of their high accuracy and analytical stability, TLGS data provide a reliable basis for advanced statistical analyses, risk-prediction modeling, and the development of national prevention policies.

## Data Availability

The dataset presented in the study is available on request from the corresponding author during submission or after publication.

## References

[1] Kim SM, Choi Y, Choi BY, Kim M, Kim SI, Choi JY (2020). Prospective cohort data quality assurance and quality control strategy and method: Korea HIV/AIDS Cohort Study. Epidemiol Health.

[2] Armstrong BG (1998). Effect of measurement error on epidemiological studies of environmental and occupational exposures. Occup Environ Med.

[3] Kirwan JA, Gika H, Beger RD, Bearden D, Dunn WB, Goodacre R (2022). Quality assurance and quality control reporting in untargeted metabolic phenotyping: mQACC recommendations for analytical quality management. Metabolomics.

[4] Chambless LE, McMahon R, Finch A, Sorlie P, Heiss G, Lyles R (1993). ARIC hemostasis study--III. Quality control. Atherosclerosis Risk in Communities. Thromb Haemost.

[5] Cushman M, Cornell ES, Howard PR, Bovill EG, Tracy RP (1995). Laboratory methods and quality assurance in the Cardiovascular Health Study. Clin Chem.

[6] Tsao CW, Vasan RS (2015). Cohort Profile: The Framingham Heart Study (FHS): overview of milestones in cardiovascular epidemiology. Int J Epidemiol.

[7] Azizi F, Zadeh-Vakili A, Takyar M (2018). Review of rationale, design, and initial findings: Tehran Lipid and Glucose Study. Int J Endocrinol Metab.

[8] Rahmani M, Jeddi S, Ghanbari M, Momenan AA, Azizi F, Ghasemi A (2019). Reference values for serum lipid profiles in Iranian adults: Tehran Lipid and Glucose Study. Arch Iran Med.

[9] Zorbozan O, Zorbozan N (2022). Evaluation of preanalytical and postanalytical phases in clinical biochemistry laboratory according to IFCC laboratory errors and patient safety specifications. Biochem medica.

[10] Alegre E, Varo N, Fernández-Calle P, Calleja S, González Á (2022). Impact of ultra-low temperature long-term storage on the preanalytical variability of twenty-one common biochemical analytes. Clin Chem Lab Med.

[11] Gruber L, Hausch A, Mueller T (2024). Internal Quality Controls in the Medical Laboratory: A Narrative Review of the Basic Principles of an Appropriate Quality Control Plan. Diagnostics (Basel, Switzerland).

[12] Wang W, Zhang Z, Zhang C, Zhao H, Yuan S, Liu J (2024). Evaluation of Coefficients of Variation for Clinical Chemistry Tests Based on Internal Quality Control Data Across 5,425 Laboratories in China From 2013 to 2022. Ann Lab Med.

[13] Hawkins R (2011). Managing the Pre- and Post-analytical Phases of the Total Testing Process. Ann Lab Med.

[14] Vani K, Sompuram SR, Naber SP, Goldsmith JD, Fulton R, Bogen SA (2016). Levey-Jennings Analysis Uncovers Unsuspected Causes of Immunohistochemistry Stain Variability. Appl Immunohistochem Mol Morphol AIMM.

[15] Peng P, Peng X, Jiao X, Chen N (2022). A unique Levey-Jennings control chart used for internal quality control in human papillomavirus detection. Virol J.

[16] Whitney CW, Lind BK, Wahl PW (1998). Quality assurance and quality control in longitudinal studies. Epidemiol Rev.

[17] Lopez J (2013). Tietz Textbook of Clinical Chemistry and Molecular Diagnosis (5th edition). Vol. 28, Indian Journal of Clinical Biochemistry.

[18] Balık AR, Gücel F (2021). Evaluation of 20 clinical chemistry and 12 immunoassay analytes in terms of total analytical error and measurement uncertainty. Scand J Clin Lab Invest.

[19] Verma M, Dahiya K, Ghalaut VS, Dhupper V (2018). Assessment of quality control system by sigma metrics and quality goal index ratio: A roadmap towards preparation for NABL. World J Methodol.

[20] Çakmak O (2018). Evaluation of analytical performance specifications of routine clinical biochemistry tests with biological variation- based total allowable error criteria. Int J Med Biochem.

[21] Lee SG, Chung HJ, Park JB, Park H, Lee EH (2018). Harmonization of laboratory results by data adjustment in multicenter clinical trials. Vol. 33, Korean Journal of Internal Medicine.

[22] Ng DK, Patel A, Cox C (2023). Data quality control in longitudinal epidemiologic studies: conditional studentized residuals from linear mixed effects models for outlier detection in the setting of pediatric chronic kidney disease. Ann Epidemiol.

[23] Røraas T, Petersen PH, Sandberg S (2012). Confidence intervals and power calculations for within-person biological variation: effect of analytical imprecision, number of replicates, number of samples, and number of individuals. Clin Chem.

[24] Parvin CA (2017). What's New in Laboratory Statistical Quality Control Guidance? The 4th Edition of CLSI C24, Statistical Quality Control for Quantitative Measurement Procedures: Principles and Definitions. J Appl Lab Med.

[25] World Health Organization (2011).

[26] Klee GG, Westgard JO (2012). Quality Management in Tietz Textbook of Clinical Chemistry and Molecular Diagnostics. Tietz Textbook of Clinical Chemistry and Molecular Diagnostics.

[27] van Rossum HH (2022). Technical quality assurance and quality control for medical laboratories: a review and proposal of a new concept to obtain integrated and validated QA/QC plans. Critical reviews in clinical laboratory sciences.

[28] Hartung ML, Baber R, Herpel E, Specht C, Brucker DP, Schoneberg A (2021). Harmonization of biobank education for biobank technicians: Identification of learning objectives. BioTech.

